# State-Of-The-Art and Recent Advances in Quantification for Therapeutic Follow-Up in Oncology Using PET

**DOI:** 10.3389/fmed.2015.00018

**Published:** 2015-03-23

**Authors:** Thomas Carlier, Clément Bailly

**Affiliations:** ^1^Nuclear Medicine Department, University Hospital, Nantes, France; ^2^CRCNA, INSERM U892, CNRS UMR 6299, Nantes, France

**Keywords:** nuclear medicine, PET, follow-up, oncology, quantification

## Abstract

^18^F-fluoro-2-deoxyglucose (^18^F-FDG) positron emission tomography (PET) is an important tool in oncology. Its use has greatly progressed from initial diagnosis to staging and patient monitoring. The information derived from ^18^F-FDG-PET allowed the development of a wide range of PET quantitative analysis techniques ranging from simple semi-quantitative methods like the standardized uptake value (SUV) to “high order metrics” that require a segmentation step and additional image processing. In this review, these methods are discussed, focusing particularly on the available methodologies that can be used in clinical trials as well as their current applications in international consensus for PET interpretation in lymphoma and solid tumors.

## 1 Introduction

Positron emission tomography (PET) with ^18^F-FDG plays a major role in the assessment of therapy response and in patient follow-up for oncology applications (1–3). More specifically, PET is being increasingly used to monitor response to therapy in solid tumors (4) and in lymphoma (5). Furthermore, PET imaging is often considered as a quantitative imaging technique since it offers the possibility of measuring *in vivo* the radiopharmaceutical concentration expressed in Bq/mL. As a consequence, one may benefit from this quantitative information to obtain metrics that may enhance (or probably replace) the visual interpretation that is still widely used in everyday clinical practice (6). In an interesting review, Tomasi and colleagues (7) advocate the use of quantitative metrics in PET for two main reasons: (i) those metrics are less user-dependent, calculated semi-automatically, and allow multi-center trials if acquisition and reconstruction parameters are carefully chosen (8, 9), and (ii) the development of novel radiopharmaceuticals targeting relevant biomarkers (10) imposes the use of an optimal quantitative approach as conventional quantitative metrics (including visual analysis) may not always be adapted for extracting relevant information. Additionally, beyond the usefulness of quantitative imaging for therapy response or prognosis, those metrics are expected to play a pivotal role for tumor characterization in line with the development of personalized medicine.

This short review provides an overview of the current use of quantitative metrics and discusses promising methodological developments in the context of therapy response and patient follow-up using ^18^F-FDG PET imaging. For this purpose, this review is divided into three sections. The first section is dedicated to a brief description of the main issues of quantitative metrics that are being used in clinical studies. It focuses on quantitative methodologies that have been already investigated and assessed for therapy response and patient follow-up. The ideas developed in this section can be seen as the depiction of “a perfect world,” given that the limits and usefulness of such quantitative approaches can be fully understood without being necessarily implemented in routine practice. Some of those metrics are promising tools while others are already employed clinically. The second section discusses the use of quantification for treatment monitoring and response-adapted therapy in lymphoma from a more clinical point of view. It is now well-established that ^18^F-FDG PET has great value for monitoring therapy and a tremendous international effort has resulted in an unified interpretation criteria in lymphoma (11). It is impressive that quantitative metrics’ role gains more and more importance in these regularly updated consensual criteria in lymphoma. In this respect, the use of ^18^F-FDG PET in lymphoma can be considered as an “almost perfect world” as far as quantitative approaches are clinically relevant for assessing therapy response in lymphoma. In contrast, up to now, there is no international consensus in using PET-based quantitative metrics for assessing therapy response in solid tumors (third section). The most recent attempt to standardize interpretation criteria has been proposed by Wahl and colleagues with the PET response criteria in solid tumors (PERCIST) (12), and paves the way toward an unified approach in solid tumors that is not yet used clinically.

## 2 Metrics for Quantification in PET: A Perfect World

Quantitative metrics derived from PET images are now recognized as valuable tools to improve the robustness of diagnosis especially in the area of therapeutic follow-up. The standardized uptake value (SUV) is now the most popular metric routinely used and is included in 90% of PET reports (13). However, other PET-derived quantitative metrics have emerged to be potentially useful in analyzing PET images or helping nuclear medicine specialists to diagnose patients with confidence. The aim of this section is not to discuss the technical limitations of all quantitative metrics but to highlight the assessment and use of those metrics under clinical situations. The metrics that can be derived directly from reconstructed volume without post-processing are termed hereafter “first order metrics”. SUV_max_ and SUV_peak_ are included in this category and are briefly detailed in the first part. “Second order metrics” fall under the category of those measurements that in addition to “first order metrics ” require a segmentation step to be computed, and include SUV_mean_, total lesion glycolysis (TLG), and the associated metabolic tumor volume (MTV). These are outlined in the second part. In the third part, “high order metrics” that require a segmentation step and additional image processing are briefly detailed. Tumor textural features are typically included in those metrics. Lastly, the usefulness of a new parametric approach exploring the benefit of tracking tumor uptake changes between longitudinal examinations is presented.

### 2.1 First order metrics: SUV_max_ and SUV_peak_

The SUV is widely adopted as a surrogate of the overall net rate of ^18^F-FDG uptake. The underlying limitations of this assumption can be found, for instance, in the review of Bai and colleagues (6). The SUV is defined as the ratio between the radiopharmaceutical concentration (expressed in Bq/mL) and the decay corrected injected activity normalized by a given factor. Three main normalization factors are used: the widely used body weight (SUV_bw_ expressed in kg/mL), the body surface area (SUV_bsa_ expressed in m^2^/mL) computed with specific equations (14), and the lean body mass (SUV_lbm_ or SUL expressed in kg/mL). This latter metric is recommended by Wahl (12) when using the PERCIST criteria because of its less dependent variation on body weight especially for obese patients. A recent work discussed the use of appropriate equations for computing the lean body mass (15).

The precise description of technical variabilities of SUV is beyond the scope of this short review and has been widely discussed in the literature. Readers interested in a thorough insight can refer to several excellent studies dealing with this issue (16–20). This paper focuses on the two most used metrics: SUV_max_ defined as the SUV value of the maximum intensity voxel within a region of interest (ROI) and SUV_peak_ defined as the average SUV within a small ROI (usually, a 1-cm^3^ spherical volume). Only these properties assessed with patients’ data are reported, which represents limited studies in spite of their widespread application and description on phantoms data. Additionally, all the following studies share the strong hypothesis that both SUV_max_ and SUV_peak_ are not affected by the partial volume effect (PVE). It is well known that this hypothesis is invalidated when the lesion size is less than three times the reconstructed image resolution (21). The PVE results from the combination of the tissue fraction effect due to the point-spread function of the PET system, and the sampling effect due to the finite voxel size of the reconstructed images. An overview of partial volume corrections (PVC) can be found in a recent paper by Erlandsson et al. (22).

In a study including 26 patients with various clinical indications (23), Nahmias et al. reported the reproducibility of SUV_max_ by acquiring two PET/CT scans within 3±2 days. They concluded that the SUV variability increased when SUV_max_ increased, but contributed <0.5 (in SUV unit) in 95% of repeated studies. This indicates that an SUV change superior to 0.5 may be clinically relevant in most cases. De Langen et al. extended the study with a meta-analysis based on four studies representing 86 patients and 163 analyzed tumors (24). The main relevant conclusions reported by the authors were that if ΔSUV_max_ > 30% and ΔSUV_max_ > 2 or if ΔSUV_max_ > 25% and ΔSUV_max_ > 3 between two exams, then the SUV change can be considered as relevant (i.e., the difference is not likely an error measurement) within the 95% confidence limit. The conclusions published by Nahmias et al. (23) were thus partially invalidated or at least more restrictive.

A potential important feature of the SUV_max_ measurement is that this metric is susceptible to be strongly affected by noise due to its single-voxel determination. Lodge et al. focused on this issue analyzing data from 20 patients acquired by a phase-based respiration gated protocol (total duration: 15 min) for known or suspected malignancies in the chest or abdomen (25). Data were reconstructed in five independent phases and reproducibility was evaluated on two consecutive phases. They also studied SUV bias using different time frame lengths from 1–15 min. They reported several interesting conclusions: (i) the variability of SUV_max_ that can be attributed to image noise accounts for half of the overall variability, (ii) a ΔSUV_max_ < 30% is still within the uncertainty of repeated measurement, and (iii) a positive bias of SUV_max_ can be as high as 30% for short acquisition time (i.e., high noise level), evaluated as 1 min per bed position for the system used in their study. The authors also reported the properties of SUV_peak_ in this work. As expected, they found that SUV_peak_ was less biased than SUV_max_ (positive bias of 10% for the same 1-min acquisition per bed position), and the impact of noise was two times less for SUV_max_. They also concluded that SUV_peak_ was not greatly affected by the voxel size (that is directly related to image noise for a same number of counts recorded). This last conclusion may be of particular interest as a recent study suggested that lesion detectability could be improved using small voxel size (typically, 2 mm × 2 mm × 2 × mm) (26), or for multicenter studies, in which different voxel size can be used. However, as mentioned by Lodge et al., it is worth noting that SUV_peak_ is likely more sensitive to PVE than SUV_max_. Additionally, Vanderhoek et al. raised the important issue of the SUV_peak_ computation as many authors used their own ROI definition for calculating this metric (27). Their study was based on the analysis of 17 patients that underwent 2 PET/CT ^18^F-FLT. They surprisingly found that the ROI definition alone could change the tumor response assessment in approximately half the population studied when choosing the response threshold proposed by PERCIST (±30%). Their conclusions underline the need to use a unique ROI definition for computing the SUV_peak_ such as the one proposed by Wahl (12): a fixed 1-cm^3^ spherical ROI centered on the high-uptake part of the tumor (which does not necessarily embed the SUV_max_ value).

Finally, although not directly related to SUV_max_ or SUV_peak_ measurements but more generally with first order metrics, we report the recent study of Boktor et al. that aimed at assessing the intrapatient variability of SUV measured in the liver (28). This is a relevant topic as there is an increasing interest to extend visual analysis to semi-quantitative analysis especially in the area of interim ^18^F-FDG response in lymphoma where the liver is often considered as a reference region (29). A total of 132 patients that underwent two or more PET/CT scans were retrospectively enrolled. The reference range for SUV liver intrapatient variability was found to be [−0.9, 1.1] indicating the intrinsic limit of SUV measurement in the liver when considered as a reference organ.

### 2.2 Second order metrics: SUV_mean_, TLG, and MTV

When using SUV_max_ or SUV_peak_, all of the tumor information is reduced to the measurement within a very limited region of the tumor (a single voxel for SUV_max_). It has been speculated that taking measurements from the whole tumor may better reflect the overall tumor burden than SUV_max_ or SUV_peak_. The SUV_mean_ is the average measure of SUV within calculated boundaries of a tumor. Once this region is determined, it is straightforward to derive the metabolic tumor volume (MTV) and the product SUV_mean_ × MTV which defines the total lesion glycolysis (TLG), first introduced by Larson et al. for evaluating the response of locally advanced aerodigestive tracttumors (30). Obviously, the delineation of tumor involves the use of a segmentation approach. Only automatic methods will be briefly discussed in this section as a manual segmentation is often associated with a higher degree of variability. Automatic segmentation is directly impacted by several image properties that, theoretically, must be accounted for: (i) noise, (ii) spatial resolution (highly post-smoothing level dependent), (iii) voxel size, (iv) heterogeneity in the tumor, and (v) uptake gradient within and outside the tumor. Zaidi et al. recently published an overview of available segmentation approaches (31). The methods used for deriving MTV and the derived SUV_mean_ and TLG could be basically categorized into two groups, namely those that are:

1. available routinely in a clinical environment, from which we can distinguish:
  (a) methods that do not need a calibration,
  (b) methods that need a calibration,
2. still under development and not routinely available.

Segmentation approaches that fall under the group 1a can be SUV^n%^ where a threshold based on the percentage of the SUV_max_ is chosen (typically, n ∈ [41 − 70]) or SUV_k_ where all voxel values that are superior to SUV = k (typically, k = 2.5 or k = 3) delineate the tumor. Segmentation techniques that need a calibration (sub-group 1b) are those developed for instance by Schaefer et al. (32) or Vauclin et al. (33). Those methods are often termed as contrast-oriented and need prior calibration. In that respect, they can be considered as specific of a given PET scanner, reconstruction algorithm, and voxel size. Methods that belong to group 2 are advanced automatic methods using only the intrinsic properties of reconstructed images. They do not need a calibration phase and are currently under development and/or assessment. The most popular, as far as PET only datasets are considered, includes edge detection (34), watersheds (35), gradient-based (36), Fuzzy C-Means (37), or fuzzy locally adaptative bayesian (FLAB) (38).

The intrinsic performances of those different approaches have been extensively evaluated with phantom experiments (39–42). The different studies underlined that more advanced methods, such as those falling under group 2, allowed higher accuracy than those of group 1b or 1a (41, 42). All methods were more or less affected by physiological and imaging parameters (40). Cheebsumon et al. avoided the use of SUV_k_-based segmentation as this may lead to strong bias (40). Interestingly, few studies assessed the repeatability of different segmentation algorithms using clinical data (43–46). For example, Cheebsumon et al. retrospectively enrolled 19 patients (10 patients underwent ^18^F-FDG and 9 ^18^F-FLT) with non-small cell lung cancer (NSCLC). They were scanned twice 1 week apart. The repeatability was assessed for ten segmentation algorithms (representing all groups previously described) and different noise and spatial resolution properties in reconstructed images. They concluded that all methods performed generally equally for the test-retest variability but some tumor delineation methods are more sensitive to image noise (gradient-based and SUV_2.5_). While SUV_2.5_-based method tended to dramatically overestimate volume, contrast-oriented methods appeared to be robust enough against noise and spatial resolution properties. Hatt et al. (44) performed a similar study for patients with esophageal cancer (^18^F-FDG) and breast cancer (^18^F-FLT). The segmentation algorithm that led the smallest test-retest variability was always the techniques that rely on group 2 techniques, while the worst were those based on manual delineation.

While the limits were clearly highlighted with both phantom and clinical data, it is striking to note that numerous studies used known-biased method (i.e., SUV_2.5_) to compute MTV or TLG. An overview of the different segmentation algorithms used in the literature to assess the prognostic value of MTV or TLG for solid tumors is mentioned by Van de Wiele et al. (47) for patients suffering from squamous cell carcinoma of the head and neck, lung carcinoma, esophageal carcinoma, and gynecological carcinoma. A meta-analysis was also recently published by Pak et al. (48) for assessing the prognostic value of MTV and TLG for patients with head and neck cancer and also pointed out the pre-eminence of biased algorithms for computing MTV or TLG. Nevertheless, although the limits of the different tumor delineation techniques used in these studies were reported, most of the studies showed that, whatever the segmentation algorithm used, a higher MTV or TLG in head and neck cancer is associated with a higher risk of adverse events or death (48). The conclusions were almost identical when considering the review published by Van de Wiele (47). MTV and TLG calculation based on basic algorithms (threshold-based or *hrmSUV_k_*-based segmentation) succeeded in correctly predicting outcome and were found to be a relevant and independent prognostic biomarker for survival.

It is interesting to note that in the last 2 years, there has been a growing interest in assessing the prognostic value of MTV and/or TLG for solid tumors. For instance, MTV defined by threshold-based algorithms were found to be associated with progression-free survival (PFS) and overall survival (OS) in salivary gland carcinoma (49). Another study showed that MTV and TLG computed with the SUV_2.5_ technique provided useful prognostic information for patients suffering from pancreatic cancer with curative intent (50). TLG computed with a threshold-based algorithm (40% of the SUV_max_) was also an independent prognostic factor for disease progression in epithelial ovarian cancer (51).

There is also a recent tremendous effort to compare the results of MTV, as computed with PET reconstructed images, with the MTV measured after tumor resection. Hatt et al (52) compared four segmentation algorithms with pathological findings (measuring the maximum diameter of the tumor) after lobectomy for 17 patients suffering from NSCLC. They underlined that in a case where tumors tended to be very heterogeneous, all delineation algorithms underestimated the maximum diameter. This result advocates the use of advanced segmentation approaches (group 2) to account correctly for uptake heterogeneity. Zaidi et al. enrolled seven patients suffering from pharyngolaryngeal squamous cell carcinoma (53). Ten PET segmentation methods were compared to surgical specimens after total laryngectomy. The surgical specimens were frozen, cut, and then digitized. This enabled a remarkable 3D-reconstruction to be compared directly with the results based on PET segmentation. Their main findings were that advanced segmentation methods (Fuzzy C-Means-based algorithm) and adaptive thresholding techniques (sub-group 1b) gave the best approximation of volumes measured on surgical specimens. A study conducted by Schaefer et al. (54) after lobectomyand mediastinal lymph node dissection in the context of lung cancer yielded the same conclusions for an adaptive-based thresholding technique.

While MTV and TLG have proven to provide useful prognostic metrics in essentially solid tumors when computed on the primary lesion, many authors suggest that a “whole-body metabolic burden” may best reflect the stage of the disease. This idea has been put forward recently with two interesting editorial commentaries related to evaluation of treatment response in hematological disease (55, 56). This approach was successfully assessed in 19 patients with non-Hodgkin’s lymphomas (NHL) by Berkowitz and colleagues (57). The authors pointed out the potential superiority of whole-body-based metrics over conventional indices in managing NHL patients. Following this, Fonti and colleagues found that total MTV computed with the threshold-based algorithm (40% of the SUV_max_) was predictive of survival (PFS and OS) in multiple myeloma patients in a retrospective study including 47 patients (58). Similar results were recently reported by Sasanelli et al. (59) for 114 patients with diffuse large B-cell lymphoma (DLBCL). They also used a threshold-based algorithm (41% of the SUV_max_) and found in multivariate analysis that total MTV was the only independent predictor of OS. In a study focusing on 59 patients with hodgkin lymphoma (HL), Kanoun et al. (60) also showed that baseline total MTV (computed with 41% of the SUV_max_) was predictive of patients outcome for PFS. Interestingly, they showed that when combining the baseline total MTV and ΔSUV_max_ between initial and interim PET, an identification of three subsets of patients with different outcomes could be derived. They highlighted the important benefits of such categorization for tailoring therapeutic strategies in HL patients and strengthened the interest of interim PET analysis with a quantitative approach. The sum of TLG for all lesions was also investigated in a study of Kim and colleagues for 140 patients diagnosed with DLBCL (61). They used a threshold-based algorithm (50% of the SUV_max_) and found that the sum of TLG was highly predictive of survivals for both PFS and OS. However, as pointed out by Basu et al. (56), the use of a whole-body metric involving TLG could be highly dependent on the severity of PVE. Not accounting for PVE for small lesions could dramatically underestimate the total TLG making the validity of this metric questionable.

Therefore, there is an acute need for defining a robust delineation method, associated with PVC when required, that makes a reliable extraction of those second order indices possible (62).

### 2.3 High order metrics: Textural features

A new class of metrics has recently emerged in PET imaging and is currently being clinically investigated. Those metrics intend to quantify the heterogeneous intra-tumoral uptake which must in turn be correlated with clinical outcome. They are calculated on reconstructed images and are often referred to as “textural features.” The image texture characteristic is not new, and was originally proposed in the early 1970s by Haralick (63). The underlying concept relies on a possible direct relation between heterogeneity at the cellular and macroscopic levels which in turn remains still unclear (64, 65). Biological heterogeneity of a tumor is conventionally associated with different histological features such as metabolism, proliferation, necrosis, vascular structure, degree of hypoxia. These properties may greatly affect the prognosis and the treatment response. Therefore, extracting textural features directly at the macroscopic level may be of great importance for personalized management of disease.

The computation of heterogeneity in medical imaging has been already applied in a wide variety of indication for several imaging modalities. Interested readers are referred to the recent review of Davnall et al. (66). In PET imaging, textural feature analysis is mainly based on statistical approaches (67). Several steps are required including: (i) tumor segmentation, (ii) derived ROI content resampling (using typically 32, 64, or 128 discrete values), (iii) desired matrix computation (cooccurrence matrix, gray-level run length matrix, neighborhood gray-level different matrix, or gray-level zone length matrix), and (iv) associated textural indices computation. Ideally, the number of resampling values must be reported to avoid misinterpretation.

Galavis et al. (68) investigated the intrinsic performances of textural indices by comparing several metrics to each other and derived a set of indices that are the most independent from matrix size and reconstruction parameters. Several other recent studies reported the significance and robustness of texture metrics using clinical data. Tixier et al. (69) studied the reproducibility of 25 indices using two PET scans acquired within 4 days. They considered 16 patients with esophageal cancer and lesions were delineated with the FLAB algorithm. Only three of their indices were robust and reproducible enough between the two scans (namely: entropy, homogeneity and dissimilarity). The impact of PVC and different tumor delineation were also investigated by Hatt et al. (70) for eight textural metrics. They found that heterogeneity parameters were more dependent on the segmentation algorithm than PVC. Although a significant absolute difference was found as a function of tumor delineation, they also concluded that this difference does not change the predictive value of each parameter (at least for entropy, homogeneity and dissimilarity). The robustness of textural indices with respect to the number of discrete values used for the resampling and the tumor delineation algorithm was also investigated by Orlhac et al. (71) using 28 patients (for a total of 188 tumors) with metastatic colorectal cancer, NSCLC, and breast cancer. They argued that at least 32 gray levels are mandatory and found that only 17 indices out of the 31 studied are robust enough against the segmentation algorithm. Only one study assessed the correlation between heterogeneity evaluated numerically and visually (72). They found a moderate correlation (0.4 < r < 0.6) and underlined the poor inter-observer agreement for the visual assessment of heterogeneity. These results strengthen the need for numerical computation of textural features.

Additionally, controversy remains regarding the number of voxels used for computing reliable textural features (i.e., not dependent on the number of voxels used). Very few authors mentioned this crucial information in their studies. Brooks and Grigsby recently published an interesting study (73) based on 70 cervical cancer tumors. They showed that for a specific metric (entropy), a minimum number of voxels was required (700 voxels) to minimize the dependence with the number of voxels. Another study speculated that the minimum number of voxels must be larger than 3 cm^3^ (72) without precisely justifying this value. Orlhac et al. (71) suggested that the limit must not be less than 4 × 4 × 4 = 64 voxels and must also take into account the spatial resolution of the PET system (at least three times the measured full width at half maximum). It is worth noting that this methodological aspect must be carefully investigated in future studies.

Another matter for debate is the potential correlation of textural indices with each other and with first or second order metrics described in Sections 2.1 and 2.2 (65). The idea behind this issue is the real additional value brought by textural features with respect to other metrics. Several studies were focused on this problem (70, 71, 74–76). In the work of Orlhac et al. (71), the correlated indices were classified in a same group. The authors succeeded in bringing up several groups of independent texture metrics. The first and second order metric were obviously highly correlated with each other but poorly correlated with the majority of textural metrics. They also focused on the correlation of MTV with textural features and found that some texture indices were strongly correlated with MTV. They concluded that this correlation must be accounted for when using such indices. Hatt et al. (70) came to the same conclusions for a more limited number of textural features.

Whilst the significance and robustness of textural features are not currently fully understood or investigated (65), there is a growing interest for using those metrics in a clinical setting. Studies were dedicated mainly to solid tumors. Tixier et al. (77) reported that textural analysis can differentiate three groups of patients (non-responder, partial-responder, and responder) with a very good sensitivity for 41 patients with esophageal cancer before external radiotherapy and chemotherapy. They showed that few textural metrics performed better than any SUV-based measurements. Cheng et al. (78) also confirmed that one of their analyzed textural metrics, uniformity calculated with the cooccurrence matrix, was an independent prognostic factor for PFS and OS for 70 patients with advanced T-stage oropharyngeal squamous cell carcinoma. In another interesting study, Cook et al. (79) retrospectively enrolled 53 patients with NSCLC and found that three textural metrics can better stratified patients treated with radiochemotherapy than SUV parameters, MTV, or TLG. Two studies recently addressed the use of textural features in response assessment (80, 81). Yang et al. (80) were interested in the temporal evolution of 22 textural metrics during the course of treatment of 20 patients with cervical cancer (three PET/CT scans). They concluded that textural features may be considered as an alternative to SUV changes for better understanding the tumor response. Bundschuh et al. (81) assessed three textural metrics in 27 patients that underwent 3 PET/CT scans in the context of locally advanced rectal cancer treated by neoadjuvant chemotherapy. The coefficient of variation metric was highly correlated to histopathologic response and could predict the disease progression better than any conventional parameters (SUV_max_, SUV_mean_, MTV, or TLG).

Finally, it is worth noting that a better understanding of biological basis of textural features is crucially needed. Multicenter studies must be also be conducted in the future to assess the robustness of textural metrics and associate textural analysis with genomics studies (82). Additionally, the association of textural features with conventional parameters may represent a good opportunity to better stratify patients and trend toward a personalized management of disease (72).

### 2.4 Parametric imaging: Nuclear medicine specialist’s best friend?

The therapeutic response assessment with PET imaging is currently based on tumor uptake change by measuring only one value. This value may reflect the change of a small number of voxels (one voxel for SUV_max_) within the tumor as discussed in Section 2.1, or the whole tumor using metrics mentioned in Sections 2.2 and 2.3. None of these approaches take into consideration the heterogeneity of change within the tumor on a voxel-per-voxel basis. These local changes may reflect a heterogeneous response of the tumor or the development of a necrotic area for example.

**Figure 1:**
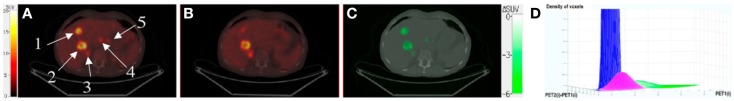
**(A)** PET1 showing five tumors, superimposed with CT1. **(B)** PET2 superimposed with CT2. **(C)** Parametric image (superimposed with CT1) showing only voxels with significant tumor changes between PET1 and PET2. These voxels are shown in green, meaning that SUV decreased between the two scans. For the two biggest tumors, the EORTC-based approach found a responding lesion (SUV decrease of 27% for tumor 1) and a stable lesion (SUV decrease of 10% for tumor 2). Parametric imaging found two responding lesions (ΔSUV = −5.9 and −2.6 for tumors 1 and 2, respectively), which were consistent with RECIST classification derived from late CT. **(D)** Biparametric graph fitted by the gaussian mixture model, for which three clusters can be distinguished: noise (blue), physiologic changes (pink), and tumor changes (green). This research was originally published in *Journal of Nuclear Medicine*. Necib et al. (83). ©by the Society of Nuclear Medicine and Molecular Imaging, Inc.

**Figure 2:**
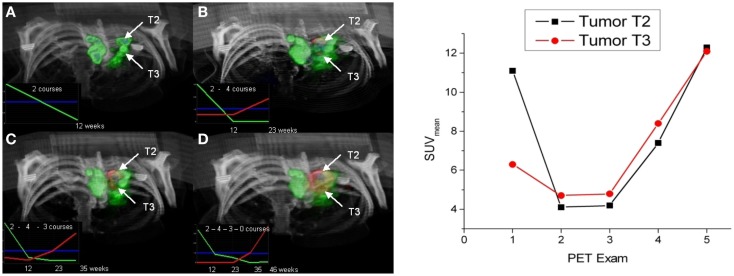
**Left**: 3D visualization of two tumors (T2 and T3) using parametric imaging with three basis functions: stable (blue), decreasing (green), and increasing (red) represented in the left corner of each image (the number of chemotherapy courses between each PET exam is mentioned). Parametric imaging using 2 **(A)**, 3 **(B)**, 4 **(C)**, and 5 **(D)** exams. **Right**: SUV_mean_ evolution (calculated within a ROI defined by 40% of SUV_max_) for the two tumors. Note that the non-responding T2 tumor was detected with parametric imaging earlier (exam 2) than applying EORTC criteria that concluded to a stable disease between exam 2 and 3. Reprinted by permission of Necib (Ph.D Thesis).

A method that takes benefit of significant intratumoral evolution was recently proposed by Necib et al. (83) using parametric imaging. This approach relies on the difference of SUV between two PET scans at a voxel level. The local changes are identified by a Gaussian mixture model. The authors applied this methodology to 78 pairs of tumor images acquired at baseline and follow-up for 28 patients with metastatic colorectal cancer. They found that their approach correlated well with RECIST and performed better than the European organization for research and treatment of cancer (EORTC) criteria when RECIST is considered as the gold standard. An example of images yielded by the parametric approach is illustrated in Figure 1. The parametric imaging approach can be extended to more than two PET exams using factor analysis. In this approach, each voxel evolution is modeled by a weighted sum of two or three basis functions representing respectively a stable, decreasing and/or increasing trend. This methodology is currently under investigation and is shown in Figure 2 for illustrative purposes.

Another approach proposed by David et al. (84) uses paradoxical theory. This approach models imprecision, uncertainty, and conflict between sources. It can be applied to two PET exams and was found to result in more consistency for partial responders than using conventional methodology that involved SUV or MTV change.

## 3 PET Scans for the Management of Lymphoma: An Almost Perfect World

The use of ^18^F-FDG PET for evaluation of HL and NHL has increased dramatically during the last decade both for staging and response assessment. Concerted efforts have been made to standardize practice and according to Cheson’s recommendations recently published (11), ^18^F-FDG PET should be realized at initial staging in all FDG-avid lymphoma histologies. Moreover, since the International Harmonization Project (IHP), which first published guidelines for the application of ^18^F-FDG PET in lymphoma in 2007 (85), international consensus recommendations for uniform PET interpretation criteria are regularly updated based on published PET literature (11, 86–88). In this scenario, the Lugano recommendations validated the use of the visual Deauville scale for response assessment in all histological FDG-avid types of lymphoma. Nevertheless, some data suggest that quantitative metrics could be used to improve visual analysis for response assessment in lymphoma and thus, metrics such as SUV_max_ have been fully integrated in recent standardized response criteria used in clinical trials.

In the 1990s, the first reports of semi quantitative measures in lymphoma staging demonstrated that the degree of uptake was largely dependent on the histology of lymphoma (89). In 2005, Schöder et al. showed that the different levels of ^18^F-FDG uptake between low-grade and aggressive lymphomas on metabolic imaging could be considered as a useful tool for assessing the transformation of a low-grade lymphoma to a more aggressive disease (90). Based on these conclusions, a prospective study was carried out to assess the value of ^18^F-FDG PET for guiding biopsies in patients with low-grade lymphoma and with clinical, radiological, or biological signs of aggressive transformation (91). This study confirmed that ^18^F-FDG PET can be used as an accurate guide for biopsies in suspected transformed tissues: a SUV_max_ < 11.7 was always associated with indolent lymphoma, whereas a SUV_max_ > 17 was always associated with histological transformation. Moreover, the ^18^F-FDG uptake gradient, observed on metabolic imaging recorded at initial DLBCL staging, could suggest transformation of unidentified low-grade lymphoma patients. Multiple authors have also studied the predictive prognostic value of early ^18^F-FDG on the outcomes of patients. If the Lugano recommendations validated the use of the visual Deauville scale for response assessment, some data suggested that quantitative metrics could also be used to improve visual analysis. The contribution offered by the development of SUV_max_ has been a great step forward and particularly investigated in DLBCL. Lin et al. were the first, in this histological subtype of lymphoma to measure the reduction of SUV_max_ in the “hottest” lesion before and during treatment, referred to as ΔSUV_max_ (92). This continuous variable may represent the dynamic process of tumor log-kill more accurately than a visual scale or a SUV cut-off value. As discussed above, SUV measurement is affected by numerous factors and therefore, it seems difficult to rely on one single SUV at a given time point to appreciate the therapeutic response and to predict outcome. Indeed, the measurement of an inter-scan SUV reduction performed under identical conditions within the same institution is probably a better and more reproducible approach. Despite the intrinsic limitations of SUV, when measured rigorously, it provides a reasonably reproducible measure of uptake that can be used to objectively assess changes related to the tumors only. This is confirmed by the published data which suggest that ΔSUV_max_ predicts outcome better than visual assessment in DLBCL in terms of progression-free survival and with better interobserver reproducibility (92–96). The optimal threshold to discriminate between good and poor treatment response groups varies between studies with cut-offs ranging from 66 to 91%, suggesting that consistency in scanning protocols and timing are mandatory for general application. Recently, the role of SUV_max_ reduction was also explored in HL with minimal residual uptake that was regarded as equivocal for the presence of disease. In a study by Rossi et al., ΔSUV_max_ was more accurate than visual analysis based on the Deauville criteria to predict outcomes of patients with HL (97), and was thought to be more particularly useful in patients with Deauville scores 3–4 in order to characterize the significance of the minimal residual uptake. Hasenclever et al. also described the use of semi-automatic quantification for interim ^18^FDG-PET response in HL (29). The authors methodology named qTEP extended Deauville scoring to a continuous scale by translating visual categories into thresholds. Yet, as seen before, the use of quantitative metrics rather than visual grading in HL is actually subject to controversy and requires further study. This could be explained by the difference in the cellular architecture and physiological features between HL and aggressive NHL. In HL, neoplastic cells account for <1% of the overall cellularity of the neoplastic tissue, whereas in NHL, they contribute more than 90% of the total cell population. In HL, non-neoplastic lymphocytes produce a cytokine network that ensures the immortalization of the neoplastic cells and works as an amplifier of the PET detection power. This non-neoplastic cellular compartment is switched-off very early by chemotherapy. On the other hand, in DLBCL, a progressive fraction of neoplastic cells are lysed by the chemotherapy, and the percentage of the cell destruction is predictive of the final response to the chemotherapy. For these reasons, a visual assessment seems preferable in HL, whereas a quantitative approach by SUV_max_ measurement seems more appropriate in DLBCL.

Because even SUV_max_ is, for a number of reasons described previously, not a reliable metric, other quantitative metrics have been proposed, including MTV or total TLG. In previous analyses, a variety of pre-therapy clinical markers were consistently associated with outcome in lymphoma patients. For example, in NHL, several patient characteristics were analyzed to determine whether they were associated with survival, and the factors that emerged as significant were, in addition to the Ann Arbor stage: age, elevated serum lactate dehydrogenase (LDH), performance status, and number of extranodal sites of disease. These were combined in the International Prognostic Index (IPI), a clinical tool developed by oncologists to aid in predicting the prognosis of patients with aggressive NHL (98). Some of these features reflect the tumor’s growth and invasive potential, to what is currently named tumor burden. Thus, from a clinical point of view, calculation of a global three-dimensional tumor burden with PET could be an important predictor of outcome in almost any type of lymphoma similar to disease bulk at initial presentation which has long been a known adverse prognostic factor, particularly in early stage HL (11). The prognostic value of tumor size using conventional imaging has previously been demonstrated and as functional imaging is more sensitive, it may be used to evaluate tumor burden more accurately. Several studies have evaluated baseline PET-based volume metrics but with very heterogeneous data caused by the lack of standardization on the calculation method. Song et al. evaluated the prognostic impact of MTV in stage II/III DLBCL without extranodal involvement (99), in primary gastrointestinal DLBCL (100), in extranodal T cell lymphoma (101) and in HL (102), using a fixed SUV_max_ threshold of 2.5. As seen previously, this methodology may overestimate the metabolic tumor volume especially when the background around the tumor has high activity leading to the inclusion of voxels from the background in the calculation. On the other hand, as discussed above, Kanoun et al. in HL (60), Sasanelli et al. in DLBCL (59), and Meignan et al. in both HL and DLBCL (103), all used a SUV_max_ threshold of 41%, as recommended in European guidelines. This threshold generally determines functional volumes accurately under specific imaging conditions of homogeneous activity “tumor-like” distribution with homogeneous background activity in phantom studies. In clinical practice, however, the lesions are often highly heterogeneous. When the lesion has low uptake, the volume can be overestimated if background activity is erroneously included. Moreover, in lesions with a very high SUV_max_, there might be the risk that the 41% threshold would eliminate a fraction of the volume with high SUV but a lower SUV than the threshold. Thus, even if pre-treatment MTV and TLG seem to be negatively correlated with progression-free survival in both HL and NHL, as exposed previously, more sophisticated segmentation algorithms are clearly needed.

Because of its enhanced sensitivity, PET imaging now plays a pivotal role in the management of lymphomas. The impact of quantitative measurement in the management of patients with lymphomas is currently being defined. The methodological concerns related to quantitative metrics are well-identified and studied and could be in a near future of valuable interest and chiefly PET center independent. Recent data suggest that quantitative measures such as SUV_max_ and more particularly ΔSUV_max_ could be used to improve visual analysis for response assessment. These latter have been incorporated into recent uniformly adopted response criteria for clinical trials. Recent guidelines enacted to standardize PET protocols and to ensure more reproducible analyses between scans and centers will hopefully soon lead to the full integration of these quantitation tools into daily practice.

## 4 PET Scans for the Management of Solid Tumors: Toward a Perfect World

Over the past decade, there has been an expansive growth in the use of ^18^F-FDG for solid tumors as a tool for therapy assessment in oncology. This spread of the PET technique was particularly enabled by its quantification ability based on SUV to allow the use of a reproducible metric for cancer management.

One of the initial roles of SUV was in the differentiation between benign and malignant lesions. This was especially used in indeterminate solitary pulmonary nodules where the standard approach was that nodules with an SUV_max_ <2.5 could be considered benign with enough confidence to avoid an immediate biopsy; these nodules could safely be monitored with CT. For example, Lowe et al. studied 89 patients and found a sensitivity of 92% and a specificity of 90% with an SUV threshold of 2.5. With visual assessment, sensitivity was 98% and specificity was 69% (104). This method of thresholding with an absolute value was also applied to different tissues. Vansteenkiste et al. found that the optimum SUV threshold for identifying malignant lymph nodes in non-small cell lung cancer was 4.4 (105). In pancreatic carcinoma, Delbeke et al. found an SUV threshold of 3.0 to be appropriate (106). Yet, many data discarded the use of a SUV based “magic line,” above which the malignant character can be affirmed. First of all, as outlined previously, many variables affect the measurement of SUV, limiting its accuracy and reproducibility (20, 107). Moreover, using a predefined absolute SUV value may result in the exclusion of small positive lesions because of a low SUV due to partial volume effect. Additionally, some well-differentiated tumors have low intrinsic SUV, whereas some inflammatory processes may have SUV levels higher than 2.5. Undoubtedly, the use of an arbitrary value for malignancy may give an impression of objectivity over visual interpretation. But, in practice, selection of a threshold involves assessing the trade-off between sensitivity and specificity. It could certainly be argued that very high sensitivity is appropriate because the clinical consequences of a false-negative interpretation are much more serious than those of a false-positive result. In this regard, visual analysis has been reported to be equivalent (104).

In the early 1990s, quantitative measurement of early treatment-induced changes in SUV also became an attractive tool for monitoring response to therapy. The feasibility of detecting small changes in tumor glucose metabolism quantitatively was first demonstrated in studies of neoadjuvant treatment of primary breast cancer, for which declines in ^18^F-FDG uptake were seen with each successive treatment cycle in good responder patients (108). Soon after, the comparison of pre and post treatment SUV for monitoring the effects of therapy was demonstrated to be correlated with response to treatment for advanced breast cancer (109), liver metastases from colorectal cancer (110), for colorectal cancer (111), glioma (112), and head and neck cancer (113). Thereafter, the percentage of SUV decrease (ΔSUV_max_) was recommended in 1999 by the EORTC position paper as a method to assess metabolic response of tumors with PET (114). Yet, given the limited data available at that time, the need for updated criteria and further standardization of PET response through quantitative parameters gradually increased. In this scenario, the PERCIST 1.0 criteria were drafted by Wahl et al. (12) as a framework that may be useful in daily practice and for harmonizing international studies. PERCIST can be considered as an attempt to validate quantitative and semi-quantitative approaches for metabolic treatment response assessment in which cancer responses assessed by PET is a continuous and time-dependent variable. The framework has the advantage of being easily applied, and with high reproducibility. Furthermore, it can be generalized to a wide variety of malignancies and situations and avoids the conceptual limitations associated with defining an optimal SUV threshold. PERCIST criteria include definitions of “lesion measurements at baseline,” “normalization of uptake,” “complete metabolic response,” “partial metabolic response,” “stable metabolic disease,” “progressive metabolic disease,” “overall response” and “duration of response” and pave the way toward an international consensus. Yet, in spite of these important efforts, currently only about 10 studies have used these criteria for response assessment in different types of solid tumors: colorectal (115–118), breast (119), esophageal (120), and lung (121–123) cancers. Some interesting considerations have arisen from these latter studies. In the three studies comparing EORTC and PERCIST criteria for response assessment (115, 119, 120), no significant difference was observed between both, but PERCIST criteria, because of clear definitions, was considered more straightforward to use. It is also important to point out that PERCIST definitions of response to therapy are based on the calculation of SUV normalized for the lean body mass. Unfortunately, SUV_lbm_ values are not easily reproducible because there is yet no agreement on the way in which this index should be determined as nine different predictive equations exist for calculating lean body mass. For this reason, four of the studies used modified PERCIST criteria with SUV_max_ instead (116–118, 123). Moreover, as pointed out by Maffione (117), there are limitations in the complete metabolic response assessment. For the PERCIST 1.0 version, it should be done visually, with complete resolution of ^18^F-FDG uptake in the target lesion, less than the mean liver activity, and indistinguishable from surrounding blood pool activity. Yet, in rectal carcinoma for example, ^18^F-FDG uptake within the tumor site after neoadjuvant chemo-radiotherapy may be higher than the surrounding background blood-pool levels probably due to residual inflammation, or physiological tracer washout via the intestine (117). These considerations lead to the possibility that a single definition of residual disease after therapy may not be valid for every type of tumor. This issue led to the proposal of a new set of criteria to assess metabolic response in rectal cancer called PET residual disease in solid tumor (PREDIST) (124).

On the other hand, even if ^18^F-FDG PET imaging can substantially benefit from using quantitative measures of uptake, some authors discarded the tendency to analyze imaging data by trusting quantitative parameters and cut-offs. Soon after the publication of the PERCIST criteria, Hofman discussed the advantages of pattern recognition (125). The authors believe that the experienced observer can accurately assess whether a site of increased uptake is probably tumor from knowledge of anatomy and prior observations of the distribution of FDG in normal tissues.

As discussed above, other potential quantitative parameters have also been developed to evaluate patient prognosis and assess therapeutic response in solid tumors. Among these parameters, volume-based PET parameters such as MTV and TLG are especially promising by quantifying tumor burden. Van De Wiele (47) and Moon (126) presented the available data in patients suffering from squamous cell carcinoma of the head and neck, lung carcinoma, esophageal carcinoma, and gynecological malignancies. These reviews of the literature suggested that MTV and TLG have the potential to become valuable as prognostic biomarkers, adding value to clinical staging or for assessment of response to treatment. However, the authors also highlighted the main difficulty of these approaches. As already explained, the lack of robust segmentation techniques for delineating tumor volume makes it difficult to draw general guidelines. However, significant results were observed in the area of prognostic and treatment response assessment in cancer patients even if most reported studies have included heterogeneous groups of patients presenting different disease stages receiving different chemotherapy regimens and used different methods for tumor delineation. However, further large-scale prospective studies are needed in order to confirm the validity of these parameters.

If an approach for response assessment of solid tumors is finally adopted by an international consensus, one should not forget that the expert reader has the task of making the last judgment call for imaging interpretation. Regardless of which system is used, EORTC, PERCIST, or PREDIST criteria, or even if visual interpretation is used, without contradicting the need for standardization for harmonizing PET response in solid tumors and without understating the importance of the efforts already achieved, quantitation remains a key tool but is not a substitute for thinking. In daily practice, referring clinicians expect to find a conclusion in the PET report in terms of “complete metabolic response,” “partial metabolic response,” “stable metabolic disease,” “progressive metabolic disease” rather than a simple percentage of SUV decline or qualitative terms like “mild,” “moderate,” or “severe” uptake.

## Conflict of Interest Statement

The authors declare that the research was conducted in the absence of any commercial or financial relationships that could be construed as a potential conflict of interest.
